# Supplementary material for: Splice-Junction-Based Mapping of Alternative Isoforms in the Human Proteome
Source: Cell Rep. Author manuscript; Available in PMC 2020 Jan 15. (PMC6961840; doi:10.1016/j.celrep.2019.11.026)
Supplement: 3 [file NIHMS1546469-supplement-3.zip › DF2/PXD009737/TestisGluC-3-Q10567-LRIQPGNPSCTLSLKCRAPE.pdf]

A

Predicted sequence disorder and sequence features of Q10567

Peptide: LRIQPGNPSCTLSLKCRAPE Junction: sp|Q10567|AP1B1_HUMAN|ENSG00000100280|SE2|30868|chr22|29328895|29329720|−2|r20|T1 TrNovel: FALSE

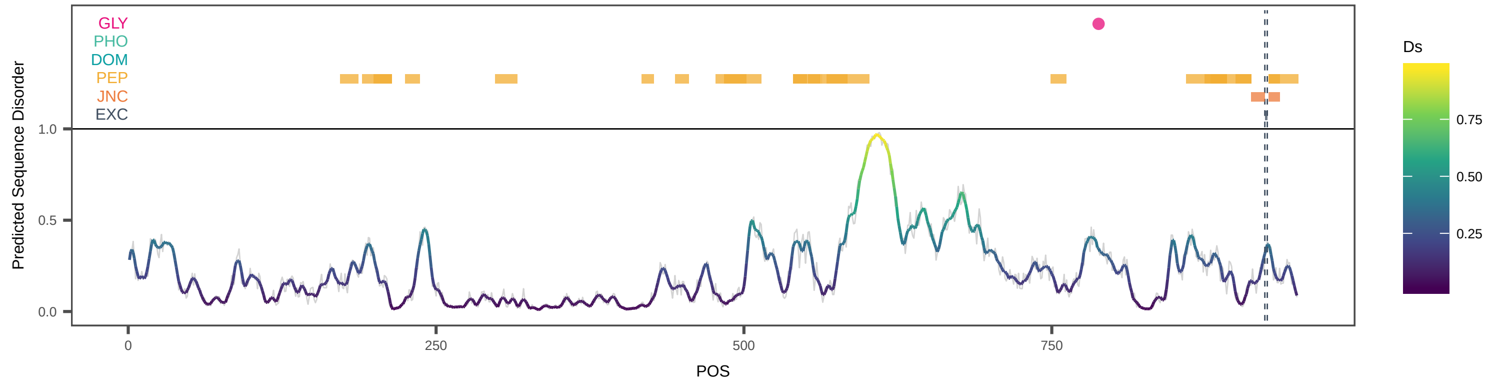

B

Distribution of sequence disorder in excised
vs. mapped and non−excised regions of protein

M−W P−value vs. mapped: 0.0876 vs. non−excised: 0.0808

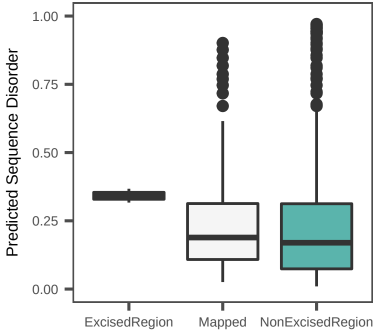

C
